# The Impact of Genetics on Craniofacial Dysplasias and Consequent Oral Malformations—Integrative Review

**DOI:** 10.3390/genes17020140

**Published:** 2026-01-27

**Authors:** Inês Lopes Cardoso, Maria Inês Guimarães, Laura Touboul, Fernanda Leal

**Affiliations:** 1 RISE-Health, Faculty of Health Sciences, Fernando Pessoa University, Fernando Pessoa Teaching and Culture Foundation, 4249-004 Porto, Portugal; 2 Faculty of Health Sciences, Fernando Pessoa University, Fernando Pessoa Teaching and Culture Foundation, 4249-004 Porto, Portugal; 3 FP-I3ID, Institute of Investigation, Innovation and Development, FP-BHS, Biomedical and Health Sciences, University Fernando Pessoa, 4249-004 Porto, Portugal; 4 Escola de Medicina e Ciências Biomédicas (EMCB), Fernando Pessoa Teaching and Culture Foundation, 4200-150 Gondomar, Portugal; 5 North Delegation—National Institute of Legal Medicine and Forensic Sciences, I. P. (INMLCF), 4099-013 Porto, Portugal; 6 CEISUC, Centre of Investigation in Technologies and Centre for Health Studies and Research, University of Coimbra, 3004-504 Coimbra, Portugal

**Keywords:** genetics, craniofacial dysplasia, cleidocranial dysplasia, ectodermal dysplasia, Apert syndrome, oral manifestations

## Abstract

Background/Objectives: Diseases affecting the craniofacial skeleton are normally associated with disturbances in the regulation of cellular differentiation, the development of bone structures, and changes in bone density and ossification. Thus, the objective of this integrative review is to evaluate the published scientific literature from the last 8 years concerning the impact of genetics on some craniofacial dysplasias. Our aim covers the identification of oral cavity alterations to those dysplasias, through the most common orofacial manifestations. Three dysplasias were selected to be part of this integrative review: cleidocranial dysplasia, ectodermal dysplasia and Apert syndrome. Methods: For this purpose, a bibliographic search was performed in the PubMed, ScienceDirect, Web of Science and Google Scholar databases with several keywords combined with each other. The research question of this review was as follows: “What is the impact of genetic factors on the development of craniofacial dysplasias and associated oral malformations?”. Results: After selecting the articles through the application of inclusion and exclusion criteria, 11 articles were selected for this review. Conclusions: Genetics plays a crucial role in craniofacial dysplasias and subsequent oral malformations. The main conclusion was that mutations in different genes can lead to identical phenotypes, while mutations in the same gene can present slight phenotypic differences depending on where they occur. In the future, it would be important to conduct studies with larger samples and control groups that include genetic testing to allow for a more comprehensive study on the impact of genetics on craniofacial dysplasias.

## 1. Introduction

Craniofacial dysplasias are generally associated with dysregulation of cell differentiation, bone pattern and development, as well as changes in bone density and ossification patterns [1].

Cleidocranial dysplasia (CD), or Marie–Sainton disease, is a rare disorder, with autosomal dominant transmission caused by mutations in the *RUNX2* gene, present in chromosome 6 (6p21). Clinically, CD is characterised by specific craniofacial and dental abnormalities associated with unilateral or bilateral aplasia or hypoplasia of the clavicles [2].

Ectodermal dysplasia (ED) also comprises a heterogeneous group of genetic disorders that affect the development of structures derived from the ectoderm, including hair, teeth, nails, and sweat glands [3]. One of the best-characterised forms is hypodrotic ectodermal dysplasia (HED). This condition results from mutations in the genes coding for ectodysplasin A (EDA), ectodysplasin A receptor (EDAR) or EDAR-associated death domain (EDARADD), being characterised by hypohidrosis, oligodontia, conical teeth, and thin hair. Anhidrotic ectodermal dysplasia (AED) involves the same genetic alterations but represents a more severe variant, characterised by the almost complete absence of sweating, leading to significant thermoregulatory challenges [4,5].

Apert syndrome, another rare craniosynostosis, is characterised by irregular craniosynostoses, hypoplasia of the midface, and syndactyly of the hands and feet. This disorder is associated with mutations in the gene encoding fibroblast growth factor receptor 2 (*FGFR2*) on chromosome 10 (q25-q26), following an autosomal-dominant inheritance pattern [6].

A multidisciplinary approach to treatment is essential for the management of patients with craniofacial disorders and dysplasias, involving the collaborative efforts of geneticists, radiologists, surgical specialists, and dentists, since most patients have multiple orofacial and dental challenges, with a possible overlap of syndromic conditions with other skeletal disorders [7].

Surgery is mainly recommended to prevent delays in neurodevelopment and the effects of elevated intracranial pressure in craniosynostosis [8]. Orthodontic treatment to correct occlusal problems and reconstruct jaws, bone grafting, and distraction osteogenesis are recommended to improve aesthetics and function [7].

While some craniofacial skeletal disorders occur in symmetrical craniofacial bones, others occur asymmetrically [9].

The head represents a complex region concerning its anatomy. Similar developmental embryology is shared by all higher vertebrates, which integrates the germ layers of the ectoderm, mesoderm, and endoderm. Cranial neural crest cells form a migratory population of multipotent stem cells. These cells interact with the germ layers to form a substantial part of the bones, cartilage, odontoblasts, and connective tissues of the craniofacial region and dental morphogenesis [10].

However, the complexity involved in integrating germ layers and precisely regulating the proliferation, differentiation, and migration of stem cells to the correct areas of the head during development often leads to craniofacial disturbances and dysplasias. Craniofacial dysplasias may be associated with lethal and non-lethal types of skeletal dysplasias, and individually, most craniofacial disorders are rare and have different patterns of inheritance [11].

Thus, the objective of this work is the analysis of scientific publications from the last 8 years on the impact of genetic factors on the development of craniofacial dysplasias and related changes to the oral cavity, through observations of the most common orofacial manifestations. Three dysplasias were selected to be part of this integrative review: CD, ED, and Apert syndrome.

### 1.1. Cleidocranial Dysplasia (CD)

CD, also known as cleidocranial dysostosis, is characterised as a genetic disorder with an autosomal-dominant inheritance pattern, with more than 40% of cases representing spontaneous mutations [12].

It is considered a rare genetic disorder, with autosomal-dominant transmission, resulting from mutations in the *CBFA1* gene, also known as *RUNX2*, present in chromosome 6 (6p21). This gene codes for a transcription factor of the *runt* family, which is responsible for controlling the differentiation of precursor cells into osteoblasts and is therefore essential for the formation of both endochondral and membranous bone tissue and for the maturation of chondrocytes. While in some families the phenotype is present due to the heterozygous loss of the CBFA1 transcription factor, in others, insertion, deletion or missense mutations cause the premature stopping of protein synthesis, affecting the DNA-binding domain or the C-terminal transactivating region [2].

The genetic alterations described lead to changes in osteoblastic differentiation, affecting bone tissue, which results in congenital anomalies in various areas of the body. The main clinical manifestations are present in the skeletal structure and oral cavity of individuals and include developmental changes in the clavicles, skull and facial bones, and teeth [13].

Among the most common characteristics of CD is the pathognomonic triad that includes aplasia or hypoplasia of the clavicles (narrow, sloping shoulders that can come close to the midline), delayed closure of the fontanelles, and the presence of supernumerary teeth [13]. Thus, in general, patients tend to present unilateral or bilateral clavicular agenesis, short stature, and hyperdontia [14,15].

When there is an incomplete presentation of these characteristics in CD, a differential diagnosis should be made with other pathologies such as Gardner syndrome, Yunis–Varon syndrome, osteogenesis imperfecta, Hallerman–Streiff syndrome, orofacio-digital syndrome type I, hydrocephalus, or pyknodisostosis [13,15].

The most relevant orofacial manifestations of this condition include bone changes such as frontal bossing, extra bones and delayed closure of cranial sutures, as well as oral cavity alterations like the delayed eruption of permanent teeth, the presence of supernumerary teeth, mandibular hypoplasia, and cleft palate [16].

However, despite the presence of all these anomalies, patients’ complaints and the main reasons they seek dental care are the presence of supernumerary teeth, delayed shedding of deciduous teeth, and the presence of unerupted teeth [2].

Radiographic examination aids in the diagnosis of dysplasia, as it allows for the identification of factors that could not be determined clinically. This radiographic examination allows for the visualisation of the unilateral or bilateral absence of clavicles, open cranial sutures, fontanelles with delayed closure, and the presence of multiple retained supernumerary teeth [13].

The main objective of treating patients with CD is to restore the functional and aesthetic pattern that is deficient. By age 5 to 7, the presence of supernumerary (extra) incisors can be clearly identified via imaging, allowing early diagnosis. Because CD often involves multiple extra teeth that block the eruption of permanent teeth, identifying them at this stage allows for a proactive surgical and orthodontic roadmap rather than a reactive one [17]. Treating the patient during this specific window addresses three critical areas before they impact long-term development: mastication, ensuring the child can chew properly to support nutritional needs; phonetics, since early dental intervention helps prevent speech impediments often associated with malocclusion or missing teeth; aesthetics, allowing for the restoration of a “normal” dental appearance before the child enters peak socialisation years in school [17,18].

Success rates are higher when treatments are performed in childhood. This is generally attributed to bone plasticity. In fact, during this stage, the alveolar bone is more adaptable to tooth movement and surgical intervention during active growth. Moreover, removing supernumerary teeth early can sometimes allow permanent teeth to erupt more naturally, reducing the complexity of orthodontic work later in life. A common barrier is the discrepancy between the ideal age for treatment and the actual age of performing it. Since CD does not typically cause systemic medical pain or life-threatening complications, many families do not seek specialised dental care until delayed eruption becomes visually obvious (often well after age 7). However, waiting until the patient is dissatisfied with aesthetics results in a more difficult clinical path than if intervention had begun earlier [18].

The proposed therapeutic strategy for these changes usually involves orthognathic surgery to correct maxillary hypoplasia, the removal of impacted teeth associated with orthodontic and/or prosthetic therapy, and the removal of retained and supernumerary deciduous teeth with malformations and/or involved in pathological lesions. However, the extraction of retained deciduous teeth alone will accelerate the eruption of permanent teeth [12,15].

The treatment protocol for manifestations focuses on the chronological and dental age of the patient, as well as the type of case and degree of intensity. According to Alves et al. [19], the most popular protocols are the Toronto–Melbourne, Jerusalem, Belfast–Hamburg, and Bronx protocols. The Toronto–Melbourne treatment strategy is based on the extraction of deciduous teeth, with supernumerary teeth also removed to facilitate the eruption of unerupted permanent teeth [20,21]. Moreover, the Belfast–Hamburg approach focuses on surgery to remove all deciduous and supernumerary retained teeth, followed by orthodontic treatment on fully erupted teeth [19,21]. The Jerusalem approach uses two surgical procedures: removal of the anterior deciduous and supernumerary teeth and orthodontic treatment; at approximately 13 years of age, the residual deciduous teeth are extracted, the unerupted canines and premolars are exposed, and evident orthodontic and surgical processes are completed [20,21]. Finally, the Bronx strategy involves the extraction of all primary and supernumerary retained teeth, followed by a second surgical phase of exposure and bonding of the impacted permanent teeth [19,21].

### 1.2. Ectodermal Dysplasia (ED)

ED is a genetic and hereditary disease characterised by an abnormality of the ectodermal structures [22,23].

Clinically, this disease is divided into two groups: X-linked recessive HED and autosomal-dominant Clouston hydrothic ED syndrome [24].

HED, also known as Christ–Siemens–Touraine syndrome, is manifested by a triad of hypohidrosis, oligodontia, and hypotrichosis. This syndrome involves structures such as the hair, nails, teeth, sweat and sebaceous glands, the epithelium, and the nervous system [25].

Facial abnormalities include a convex profile, a flat nose, and prominent dry lips [24].

The most common changes in teeth include anodontia, hypodontia, retained teeth, loss of vertical dimensions of occlusion and deformed teeth. These symptoms probably result from the poor development of the alveolar bone. In terms of shape and size, the teeth are usually small, conical, bulbous or taurodontic, and there is often a lot of space between each tooth. Studies show that the enamel frequently suffers deterioration and mechanical damage [26,27,28,29,30].

Other oral manifestations include cleft lip and palate [31].

Sometimes there may be atrophic inflammation of the oral cavity mucosa, hoarseness, and difficulty swallowing [32].

In contrast to X-linked HED, patients with Clouston syndrome have normal sweating and no changes in teeth. However, other ectodermal structures may be affected: the nails may be slow-growing, hypoplastic or dystrophic; the scalp and body hair may be hypochromic, fine, slow-growing and sparse [24].

HED is, in fact, the most severe and more frequent form, affecting between 1 and 7/10,000 births [26,27], while the prevalence of Clouston syndrome is unknown.

Prosthetic treatment is often the first line of defence, especially in paediatric patients. Early intervention is crucial for both physiological and psychological development. Early-age intervention involves implementing prosthetic solutions at a young age, assisting children in developing essential speech, chewing, and swallowing abilities. Prostheses help improve facial support and maintain proper temporomandibular joint function. Treatment focused on uniting or reshaping conic teeth improve the patient’s smile and self-esteem [31,33].

As the patient matures, more invasive surgical and mechanical treatments are used to provide permanent stability and dental alignment. Advanced surgical procedures such as bone grafts are used in cases of significant bone loss to provide a base for implants. Maxillary sinus lifts can be performed to increase bone height in the upper jaw, ensuring successful implant integration. Implants placed in the anterior portion of the mandibular arch have shown high success rates in children. These implants provide a stable foundation for permanent dental restorations. Specialised orthodontic care is used to align existing teeth, manage spacing, and prepare the mouth for implants or grafts [30,33,34].

Long-term management involves maintaining the health of the oral cavity and managing secondary symptoms of ED.

Because ED often affects salivary gland function, therapies aimed at maintaining moisture are essential to prevent discomfort and mucosal damage [30,33]. Since patients with ED are at a higher risk for dental decay, rigorous preventative protocols are implemented to preserve existing tooth structure. Ongoing monitoring ensures that chewing capacity and aesthetics remain optimal as the patient grows [30].

### 1.3. Apert Syndrome

Apert syndrome, a rare craniosynostosis, is associated with mutations in the *FGFR2* gene located on chromosome 10 (q25-q26), following an autosomal-dominant inheritance pattern [6].

This syndrome is characterised by irregular craniosynostoses, i.e., altered growth of the skull and face, leading to the elongation of the patient’s head, with a high forehead and deep-set eyes, together with abnormal closure of the eyelids. In addition, other effects of abnormal skull growth include low intellectual development, as observed in most children diagnosed with Apert syndrome, obstructive sleep apnoea, and recurrent ear infections [35].

Another phenotypic feature of Apert syndrome is syndactyly of the hands and feet [36].

There is also a wide variety of central nervous system anomalies associated with Apert syndrome, leading to the common occurrence of mental disability in patients diagnosed with this syndrome [37].

Hypoplasia of the midface is also common, leading to a concave face, and can result in the reduced volume of the nasopharyngeal and oropharyngeal spaces. This can cause chronic mouth breathing, respiratory problems, obstructive sleep apnoea, or even sudden death [38].

Altered vision can also be observed in these patients due to shallow orbits with ocular proptosis. Ophthalmological sequelae such as refractive errors, divergent strabismus, amblyopia, exposure keratopathy, and optic nerve atrophy may be observed [39].

Oral manifestations in individuals with Apert syndrome can be grouped into skeletal, occlusal, dental, and soft-tissue findings. Skeletal abnormalities include maxillary hypoplasia and a high, ogival palate, sometimes associated with cleft palate or bifid uvula. Occlusal features commonly comprise Angle Class III malocclusion, anterior open bite, and crossbite. Dental findings include crowding, delayed or ectopic tooth eruption, retained or supernumerary teeth, and other eruption disturbances. Soft-tissue manifestations include macroglossia, thickened gingiva, and inadequate lip posture [40].

Since Apert syndrome is a complex disorder, its management requires a multidisciplinary and long-term treatment strategy. During childhood and adolescence, surgical interventions are performed in coordination with dental and orthodontic care. At these stages, dental management focuses on continuous monitoring and guidance of tooth eruption, the prevention of dental caries, and early orthodontic measures to facilitate proper alignment of the teeth within the dental arches. In adulthood, patients who present with Class III skeletal malocclusion and anterior open bite secondary to maxillary hypoplasia are typically managed surgically. This treatment usually involves orthognathic procedures, such as Le Fort I osteotomy of the maxilla combined with sagittal split osteotomy of the mandible, planned in close coordination with pre- and post-surgical orthodontic treatment. Throughout all stages of care, careful monitoring of tooth eruption and alignment remains essential due to the high prevalence of dental anomalies in these patients [41].

## 2. Materials and Methods

A research protocol was established following the Joanna Briggs Institute (JBI) model [42,43,44], leading to the following research question: How do genetic factors influence the development of craniofacial and oral manifestations in Craniofacial dysplasias? To ensure structural consistency, the PICO components were defined as shown in Table 1.

For the final review, study selection was performed using guidance based on systematic reviews and meta-analysis extensions (PRISMA-ScR). In accordance with the inclusion criteria, this review specifically included case reports and case studies to ensure a detailed analysis of individual clinical presentations.

This protocol was registered with the OSF (https://osf.io/cvr52/; accessed on 28 March 2025).

### 2.1. Inclusion and Exclusion Criteria

The inclusion criteria were as follows: publications addressing the work topic conducted on humans; articles in English, French, or Portuguese published between 2018 and 2025. Eligible study designs were limited to case reports and case studies involving patients with craniofacial dysplasias.

The exclusion criteria included articles that, after reading the abstract, did not present scientific content relevant to this review; articles on animals; and articles published prior to 2018.

### 2.2. Search Strategy

The search strategy was planned by two reviewers and validated by a third using the Peer Review of Electronic Search Strategies (PRESS) checklist [45]. This integrative review included searches in PubMed, ScienceDirect, Web of Science, and Google Scholar, covering the period from 2018 to 2025. Following JBI recommendations, a preliminary search identified key terms to develop specific strategies for each database, which were executed on 27 December 2025. These detailed search strings, including Boolean operators and filters for case reports and case studies, are summarised in Table 2.

The bibliography listed in all included papers was screened for the possible inclusion of additional studies. After the search, the identified articles were deposited in the ENDNOTE programme. The results of the search were transferred to Rayyan^®^ version 1.6.0 [46], which helped with the removal of duplicates. The software developer was Rayyan Systems Inc. of Cambridge, MA, USA, and it was used to gather all the articles found in the previously described databases and to identify duplicates.

### 2.3. Analysis of Methodological Quality and Risk of Bias

The methodological quality and risk of bias of the included studies were assessed using the JBI Critical Appraisal Checklist for Case Reports [47]. This eight-item tool evaluates the clarity of the clinical presentation, diagnostic timeline, and reporting of clinical outcomes. Two reviewers independently assessed each study, categorising the overall risk of bias as low (all domains low risk), moderate (at least one domain with concerns), or high (multiple domains with concerns or critical flaws). Discrepancies were resolved through discussion or by a third reviewer to ensure a transparent and reproducible assessment.

## 3. Results

Data from the selected publications was collected by two reviewers independently to decide on their inclusion in this integrative review. Questions and conflicts were discussed with a third reviewer in accordance with the Peer Review of the Electronic Search Strategies (PRESS) checklist [45].

**Figure 1:**
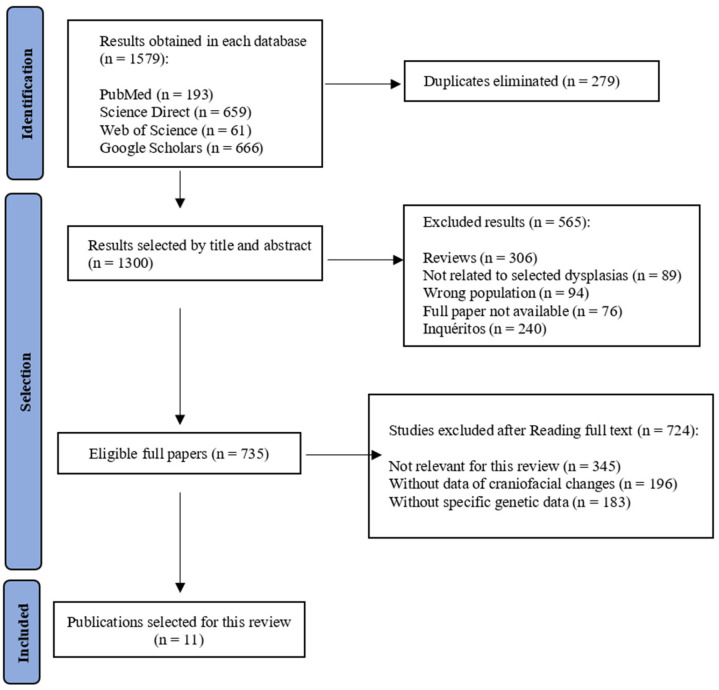
Flowchart of the PRISMA diagram adapted from PRISMA 2020 flow diagram [48].

Screening of selected publications was first carried out by taking into account the title and abstract, and in a second phase, by reading the full texts. The Prisma Flowchart present in Figure 1 shows the search methodology.

Figure 1 represents the search strategy used in this integrative review, which led to the inclusion of 11 articles, of which 3 were studies concerning CD, 5 were studies on ED, and 3 were studies on Apert syndrome.

Data were categorised by craniofacial dysplasia to facilitate analysis (Table 3, Table 4 and Table 5).

Table 6 presents an assessment of bias risk in the studies selected for this integrative review, according to the criteria of the JBI tool 2017 [47].

In general, all included studies demonstrated low risk of bias in the applicable domains (Q1–Q8). Most studies met all the relevant criteria, with some items marked as ‘not applicable’ (.), meaning they did not affect the assessment of bias.

Concerning the studies on CD, works by Gowda Venkatesha [49] and Zhang [50] demonstrate low risk of bias in the diagnostic domains (Q1–Q4) but high risk (not applicable/not reported) in domains Q5–Q7. These reports focus heavily on the molecular identification of *RUNX2* mutations and the resulting phenotypes (failed eruption/supernumerary teeth) rather than the clinical management process. From the selected studies, the study carried out by Inchigolo et al. [51] stands out as the most robust study in this group. It is the only one to fulfil Q5 and Q6, as it provides a detailed account of the surgical and orthodontic management used to treat the supplementary teeth.

Regarding ED, selected studies show a uniform reporting pattern (satisfying Q1, Q2, Q3, Q4, and Q8). There is a strong emphasis on the mutational spectrum (*EDA*, *EDAR*, *TSPEAR*). While the evidence for oligodontia and conical teeth is highly reliable due to the use of molecular biology (low bias in Q4), there is a total lack of reporting on adverse events (Q7). In terms of clinical validity, the consistency across these five studies strengthens the phenotypic certainty of the findings, despite the lack of follow-up data.

Finaly, the Apert syndrome studies focus on *FGFR2* mutations. In the study by Cammarata-Scalisi et al. [58], the patients are newborns. This naturally results in “Not Applicable” markings for Q5 and Q6 (treatment and post-intervention), as definitive dental treatment cannot be performed at that age. From the three selected studies, the study by Sikdar [57] provides a more detailed description of the Class III facial pattern and root development issues. Although it shows the same JBI score as the others, the descriptive depth reduces the risk of information bias regarding the clinical manifestations.

## 4. Discussion

This review provides two critical insights into the management of craniofacial dysplasias. First, it demonstrates that molecular genetic analysis serves as a definitive tool for differential diagnosis in cases where clinical phenotypes, such as supernumerary teeth or delayed eruption, overlap between syndromes like cleidocranial dysostosis and Apert syndrome. Second, by synthesising case-specific data, this study highlights that early genetic confirmation (between ages 5 and 7) is the primary predictor of successful orthodontic intervention, allowing for a proactive surgical roadmap that prevents the aesthetic and functional decline typically seen when patients seek care only after eruption failure. This shift from reactive to proactive management, guided by genotype–phenotype mapping, is a cornerstone for improving long-term patient outcomes [7].

In the case of CD, only three studies reporting both genetic data and orofacial findings were included. In all cases, the disorder was associated with pathogenic deletions of the *RUNX2* gene, resulting either in amino acid substitutions in the RUNX2 protein [50,51] or in the premature termination of protein synthesis due to truncated protein products [50]. These loss-of-function alterations in RUNX2, a key transcription factor regulating osteoblast differentiation and tooth development, are directly linked to the observed phenotype. Specifically, impaired RUNX2 activity disrupts intramembranous ossification, leading to delayed closure of cranial sutures and fontanelles, while its role in odontogenesis explains the characteristic retention of deciduous teeth and presence of supernumerary teeth in affected individuals. Thus, disruption of these functions explains the distinct dental anomalies associated with this disorder [16].

In the case of ED, five studies reporting both genetic data and orofacial manifestations were included, emphasising the clinical relevance of genetic heterogeneity in this condition. Three studies identified mutations in the *EDA* gene as the underlying cause of ED [52,54,55]: one study associated ED with variants in the *TSPEAR* gene [5], and another reported combined mutation in the *EDA*, *TP63* and *TSPEAR* genes [53]. From a clinical perspective, patients carrying *EDA* gene variants predominantly exhibited oligodontia [52,54], reflecting the central role of EDA signalling in tooth initiation and development. A similar oral phenotype was observed in individuals with *TSPEAR* gene variants, who also mainly presented oligodontia [5]. These findings indicate that, despite differing genetic etiologies, overlapping dental phenotypes may occur, highlighting the importance of genetic characterisation for accurate diagnosis, prognosis, and the individualised dental and orthodontic treatment planning in ED patients.

In the case of Apert syndrome, only three studies reporting both genetic data and orofacial alterations were included, and all identified mutations in the *FGFR2* gene as the etiological cause [56,57,58]. Notably, the studies by Cammarata-Scalisi et al. [58] and Sikdar et al. [57] described mutations affecting the same codon, which were consistently associated with abnormal dentition characterised by crowding, delayed tooth eruption, and the absence of root formation. Importantly, El-Bassyouni et al. [56] reported a mutation in a different *FGFR2* codon that nevertheless resulted in a similar oral phenotype. These findings refine existing knowledge by suggesting that, within Apert syndrome, distinct *FGFR2* mutations converge on a comparable pattern of dental and eruption disturbances, indicating a degree of phenotypic consistency in the oral cavity despite genotypic variability. This complements the established literature, showing that most Apert cases arise from spontaneous paternal variants at the commonly affected nucleotides c.755C>G (p.Ser252Trp) or c.758C>G (p.Pro253Arg) in FGFR2 [59]. While these canonical variants are well known for disrupting cranial bone growth and producing craniosynostosis and syndactyly, the key diagnostic features distinguishing Apert syndrome from other craniosynostosis syndromes [60], the reviewed studies extend this understanding by emphasising their predictable impact on dental development, thereby strengthening genotype–oral phenotype correlations in Apert syndrome.

Therefore, dentists play a pivotal role in first-line diagnosis. Referring the patient for genetic evaluation should be initiated immediately if the patient shows the ‘triad’ of pathognomonic oral signs: multiple supernumerary teeth (indicative of CD), severe oligodontia with conical-shaped teeth (indicative of ED), or significant maxillary hypoplasia associated with syndactyly (indicative of Apert syndrome). If performed before the age of 7, the referral is more effective, ensuring that the genetic diagnosis can guide the timing of surgical interventions and orthodontic planning.

Dental management must be multidisciplinary, integrating specialties such as orthodontics, prosthodontics, and paediatric dentistry with medical specialists, including geneticists, otorhinolaryngologists, and dermatologists. This coordinated approach ensures that the molecular diagnosis directly informs the mechanical limits of orthodontic movement and the timing of prosthetic rehabilitation. Furthermore, long-term follow-up with speech therapists and psychologists is essential to address the functional and psychosocial impacts of these dysplasias [7].

This integrative review has important limitations. Craniofacial dysplasias are rare genetic conditions, and consequently, the available literature is largely composed of case reports and small case series, resulting in limited sample sizes. This predominance of descriptive, case-based evidence restricts the ability to draw robust or generalizable conclusions and precludes meaningful statistical analysis. In addition, many studies report patients with different dysplasias without accompanying genetic characterisation, which further limits the possibility of establishing clear genotype–phenotype associations between specific genetic alterations and oral manifestations. Therefore, the findings should be interpreted with caution. Future research based on larger, systematically characterised cohorts integrating genetic and clinical data is required to confirm and strengthen these observations.

## 5. Conclusions

This review highlights that genetic mutations are the main cause of the selected craniofacial dysplasias and their related oral malformations, and different mutations can produce similar clinical features, while mutations in the same gene may result in varied symptoms.

Early genetic diagnosis enables more effective, proactive treatment and better outcomes, and multidisciplinary care, combining dental, medical, and genetic expertise, is essential for optimal management.

Dentists play a critical role in the early detection of craniofacial dysplasias and referral for genetic testing, since the associated oral manifestations significantly impact patient quality of life and functional development.

Future research should prioritise longitudinal studies with larger cohorts and standardised genetic testing to further clarify the complex genotype–phenotype correlations in these rare conditions.

## Figures and Tables

**Table 1:** PICO (population, intervention, comparison, and outcome) strategy.

Population	Individuals with Craniofacial Dysplasias (CD, ED and Apert Syndrome)
Intervention	Genetic factors/mutations
Comparison	Individuals without genetic predisposition or with different genetic predisposition
Outcome	Craniofacial and oral manifestations

**Table 2:** Strategy of the bibliographic search.

Databases	Articulation of Keywords	Number of Articles
PubMed	(((craniofacial dysplasia) OR (craniofacial abnormalities)) AND (((genetic predisposition[MeSH Terms]) OR (genetic predisposition[Title/Abstract])) OR (hereditary factors))) AND (((Oral Malformations) OR (Oral Abnormalities)) OR (Oral Developmental Disorders))	193
Science Direct	(((craniofacial dysplasia) OR (craniofacial abnormalities)) AND (((genetic predisposition[MeSH Terms]) OR (genetic predisposition[Title/Abstract])) OR (hereditary factors))) AND (((Oral Malformations) OR (Oral Abnormalities)) OR (Oral Developmental Disorders))	659
Web of Science	((craniofacial dysplasia) OR (craniofacial abnormalities)) AND (((genetic predisposition) OR (genetic predisposition)) OR (hereditary factors))) AND (((Oral Malformations) OR (Oral Abnormalities)) OR (Oral Developmental Disorders))	61
Google Scholars	TI craniofacial dysplasia OR AB craniofacial dysplasia OR SU craniofacial dysplasia OR TI craniofacial abnormalities OR AB craniofacial abnormalities OR SU craniofacial abnormalities AND TI genetic predisposition OR AB genetic predisposition OR SU genetic predisposition OR TI hereditary factors OR AB hereditary factors OR SU hereditary factors AND TI Oral Malformations OR AB Oral Malformations OR SU Oral Malformations OR TI Oral Developmental Disorders OR AB Oral Developmental Disorders OR SU Oral Developmental Disorders	666

**Table 3:** Data collected from selected CD studies.

Author [Reference]	Title	Type of Study	Sample (Age)	Results
Gowda Venkatesha et al. [49]	Clinical and radiological insights of cleidocranial dysplasia: A case report of a rare medical condition	Case report	1 male patient (25 years old)	- 11.38 kb microdeletion in the *RUNX2* gene. - Delayed closure of sutures and fontanelles. - Multiple retained deciduous teeth and multiple missing permanent teeth.
Zhang et al. [50]	A novel 90-kbp deletion of RUNX2 associated with Cleidocranial dysplasia	Case report	1 male patient (18 years old)	- Heterozygous deletion of 90 kbp in the *RUNX2* gene, causing a substitution (p.Asn183Ile) and premature termination (p.Asp184). - Markedly open fontanelles, delayed closure of cranial sutures, and maxillary dysplasia. - 14 deciduous teeth that were retained, 16 permanent teeth that failed to erupt, and 13 supernumerary teeth.
Inchigolo et al. [51]	Genetic pattern, orthodontic and surgical management of multiple supplementary impacted teeth in a rare, Cleidocranial Dysplasia Patient: A case report	Case report	1 female patient (22 years old)	- Heterozygous variant in the *RUNX2* gene (c.674 G>A, p.Arg225Gln) - Delayed closure of cranial sutures. - Immature impacted supernumerary teeth with incomplete root development.

**Table 4:** General characteristics of selected ED studies.

Author [Reference]	Title	Type of Study	Sample (Age)	Results
**Sirica et al.** [5]	Expanding the mutational spectrum of TSPEAR in Ectodermal dysplasia type 14: A familial case study	Case series	1 patient	- Two variants in the *TSPEAR* gene (c543 G>A and c1251 G>C). - Oligodontia with dysmorphic and pointed maxillary central incisors.
**Reinhold et al.** [52]	New observation of severe tooth malformation in a female patient with ectodermal dysplasia due to the EDA splice acceptor variant c.742-2 A>G	Case report	1 female patient (7 years old)	- *EDA* gene variant (c742-2 A>G). - Oligodontia. - Presence of conical teeth.
**Yapijakis et al.** [53]	Clinical and molecular genetic analysis of cases with Ectodermal dysplasia	Case series	8 patients	- Five patients hemizygous for deletions in the *EDA1* gene (Xq13.1), one patient with a heterozygous missense mutation in the *TP63* gene (3q28), and two patients with heterozygous mutations in the *TSPEAR* gene (21q22.3). -Partial anodontia of deciduous and permanent teeth. - Hypodontia.
**Zaki** [54]	Rare paediatric genetic case report of X-linked Hypohidrotic Ectodermal dysplasia type 1	Case report	1 patient	- Mutation in the *EDA* gene. - Oligodontia and multiple tooth defects. - Low salivary flow.
**Vottoru et al.** [55]	Hypohidrotic ectodermal dysplasia- A case series demonstrating indistinguishable phenotypes produced by autosomal recessive and x-linked forms	Case series	3 patients (10, 15 and 19 years old)	- Mutations in the *EDA* and *EDAR* genes. - Complete absence of teeth.

**Table 5:** General characteristics of selected studies on Apert syndrome.

Author [Reference]	Title	Type of Study	Sample (Age)	Results
**El-Bassyouni et al.** [56]	Insight into Apert syndrome: Reporting on six patients and increasing awareness	Case series	6 patients (3, 4 and 7 years old)	- Individuals heterozygous for the c758 C>G (pPro253Arg) variant in the *FGFR2* gene. - Impacted teeth, delayed eruption, and supernumerary teeth.
**Sikdar et al.** [57]	Apert’s Syndrome: A rare congenital disorder	Case report	1 male patient (8 years old)	- Point mutation of the *FGFR2* gene (c755 C>G, pSer252Trp). - Probable crowding in the upper arch and crowding and anterior rotation in the lower arch, lack of root formation in the upper and lower premolars and canines, that might indicate delayed skeletal growth and younger dental age. - Hypoplastic maxilla and zygomatic bone with normal mandible development, resulting in a concave facial pattern with a tendency towards Class III.
**Cammarata-Scalisi et al.** [58]	Clinical and genetic findings of two cases with Apert syndrome	Case Report	2 female patients (newborn and 2 months old)	- Heterozygous missense mutation (c755 C>G, pSer252Trp) in the *FGFR2* gene. - Abnormal dentition, with gaps in the eruption of deciduous teeth, delayed eruption of permanent teeth, and presence of supernumerary teeth.

**Table 6:** Methodological evaluation of articles using the JBI tool [47].

Author [Reference]	Q1	Q2	Q3	Q4	Q5	Q6	Q7	Q8
El-Bassyouni et al. [56]	√	√	√	√	.	.	.	√
Sirica et al. [5]	√	√	√	√	.	.	.	√
Gowda Venkatesha et al. [49]	√	√	√	√	.	.	.	√
Reinhold et al. [52]	√	√	√	√	.	.	.	√
Yapijakis et al. [53]	√	√	√	√	.	.	.	√
Zaki [54]	√	√	√	√	.	.	.	√
Votturu et al. [55]	√	√	√	√	.	.	.	√
Zhang et al. [50]	√	√	√	√	.	.	.	√
Inchigolo et al. [51]	√	√	√	√	√	√	.	√
Sikdar et al. [57]	√	√	√	√	.	.	.	√
Cammarata-Scalisi et al. [58]	√	√	√	√	.	.	.	√

.: Not applicable.

## Data Availability

No new data were created or analyzed in this study. Data sharing is not applicable to this article.

## Abbreviations

The following abbreviations are used in this manuscript:

AED	Anhidrotic ectodermal dysplasia
CD	Cleidocranial dysplasia
ED	Ectodermal dysplasia
EDA	Ectodysplasin A
EDAR	Ectodysplasin A receptor
EDARADD	EDAR-associated death domain
HED	Hypodrotic ectodermal dysplasia
JBI	Joanna Briggs Institute
